# Karnofsky performance scale and modified Rankin scale as indicators of functional capacity in patients with mitochondrial disease

**DOI:** 10.1007/s10072-026-09212-z

**Published:** 2026-07-15

**Authors:** Jasmin A. Koskiniatis, Mika H. Martikainen

**Affiliations:** 1 Clinical Neurosciences, University of Turku, Turku, Finland; 2 Neurocenter, Turku University Hospital, Turku, Finland; 3 Research Unit of Clinical Neuroscience, Neurology, University of Oulu, Oulu, Finland; 4 Neurocenter and Medical Research Center, Oulu University Hospital, Oulu, Finland; 5 Department of Neurology, Oulu University Hospital, P.O. Box 10, OYS, Oulu, FIN-90029 Finland; 6 Department of Neurology, Turku University Hospital, Turku, Finland

**Keywords:** Genetics, Functional capacity, Mitochondrial disease, Performance scales

## Abstract

**Background:**

Mitochondrial diseases are genetic multisystem disorders. Only symptomatic treatment is available, and clinical progression is common. We investigated whether two commonly used quantitative measures of functional capacity, modified Rankin scale (mRS) and Karnofsky Performance scale (KPS) scores, could be determined retrospectively based on electronic patient records (EPRs) and whether they provided insights into disability and disease progression.

**Methods:**

Previously identified 52 patients (28 women) with clinically and genetically confirmed mitochondrial disease at Turku University Hospital (TUH, Turku, Finland) were investigated. Genetic diagnoses were the m.3243 A > G mitochondrial DNA (mtDNA) variant (*N* = 21), other pathogenic mtDNA variants (*N* = 22), and nuclear gene variants causing mitochondrial disease (*N* = 9). Mean age was 50 years (range 10–85 years); average follow-up was nine years. Available neurology and emergency medicine EPRs were reviewed, and mRS and KPS scores determined.

**Results:**

Patients harbouring the m.3243 A > G, other mtDNA variants, or nuclear gene variants were compared. In all groups, functional capacity declined over time. Those with m.3243 A > G had lower first and latest KPS and mRS values than those with nuclear gene variants (*p* < 0.004 for all). Differences between the m.3243 A > G and other pathogenic mtDNA variants were not significant.

**Conclusion:**

Functional decline seems a common feature in mitochondrial disease. The KPS and mRS scales may offer a simple tool for long-term evaluation of the functional capacity of patients with mitochondrial disease, especially in non-specialist and primary healthcare. Further studies are needed to confirm whether patients with nuclear gene defects are at particular risk of progression.

**Supplementary Information:**

The online version contains supplementary material available at 10.1007/s10072-026-09212-z.

## Introduction

Mitochondrial disease is a common form of genetic neurometabolic disease that affects the mitochondrial respiratory chain function resulting in an impaired adenosine triphosphate (ATP) production [1]. The most common causes of mitochondrial disease in adult population are pathogenic variants in the mitochondrial DNA (mtDNA). The m.3243 A > G variant in *MT-TL1* is the most common variant causing adult mitochondrial disease. The phenotypes associated with this variant include maternally inherited diabetes and deafness (MIDD) and the mitochondrial encephalomyopathy, lactic acidosis and stroke-like episodes (MELAS) syndrome. Clinical phenotypes associated with sporadic, large-scale mtDNA deletions include (chronic) progressive external ophthalmoplegia ((C)PEO), the Kearns-Sayre syndrome (KSS), and generalised mitochondrial myopathy. Myoclonic epilepsy with ragged-red fibres (MERRF) is a clinical syndrome usually associated with the m.8344 A > G mtDNA variant. The clinical features of mitochondrial disease are, however, not restricted to definite clinical syndromes, but more narrow presentations such as mitochondrial diabetes and isolated sensorineural hearing loss are encountered too [2]. In the paediatric population, the role of pathogenic variants in nuclear genes that encode respiratory chain complex subunits or those associated with mitochondrial DNA maintenance and replication is more pronounced [3]. The latter may give rise to secondary multiple mtDNA deletions or mtDNA depletion. In mitochondrial disease associated with recessive *POLG* variants, common presentations include early-onset hepato-encephalopathy, ataxia, and severe, treatment-resistant epilepsy.

The overall prevalence of mitochondrial disease has been estimated as high as 1:4300 [2]. Recently, the prevalence of mtDNA disease in the adult population of Southwestern Finland was estimated at 9.2/100,000, and disease related to the common m.3243 A > G variant at 4.2/100,000 [4].

Since mitochondrial disease disrupts the cellular aerobic energy production, organs with high energy demand such as the brain, heart, skeletal muscle, and endocrine pancreas are commonly affected [5]. Age of onset is also variable. Severe forms of disease such as the Leigh syndrome may have onset in infancy and lead to early death, whereas in progressive external ophthalmoplegia (PEO) the disease onset is typically in adulthood or even in old age [5]. Common clinical features of mitochondrial disease include myopathy and cardiomyopathy, diabetes mellitus, sensorineural hearing loss, and epilepsy. Severe and even life-threatening features include stroke-like episodes (SLEs) and treatment resistant status epilepticus [6–8]. The m.3243 > G variant in mtDNA is particularly associated with stroke-like episodes, whereas *POLG* variants carry a higher risk of death from treatment resistant status epilepticus [7]. Pathogenic variants in *POLG* represent the leading genetic cause of mitochondrial epilepsy across all age groups and contribute to approximately 10–15% of cases of PEO (dominant variants) and more than 10% of cases with ataxia [9].

There are some previous studies on how quality of life (QoL) is affected by mitochondrial disease. Mitochondrial myopathy patients with higher disease progression scores have a worse QoL and conversely, patients with more preserved muscle strength have higher QoL [10]. In another study of 72 patients with the m.3243 A > G variant, patients with increased fatigue and mental health issues reported impaired QoL. However, associations with variant heteroplasmy levels or clinical manifestations were not strong [11]. Similarly, in a study of 95 patients with mitochondrial disease, various clinical manifestations correlated significantly with physical functioning and societal participation as well as overall QoL. However, no correlation between disease manifestations and depressive symptoms was observed [12]. Despite that several studies suggest functional deficits are common in mitochondrial patients, it is reassuring that at least in the setting of a tertiary referral centre, specialised in mitochondrial disease, patients and their carers demonstrate a high perceived level of social support [13].

Looking at specific clinical tests to assess functional ability in mitochondrial disease, one study suggested that the 10 m walk test and the timed up and go test could serve as suitable and clinically relevant clinician rated measures to track disease progression [14]. An Italian study of 118 mitochondrial disease patients found that functional measures also correlated with perceived fatigue and pain severity [15]. In a further study of 156 adult patients with mitochondrial disease, all used tests of endurance, balance, strength and mobility showed a negative deviation compared to healthy reference values. In particular, balance tests correlated with clinical severity [16].

The ability to monitor clinical progression in mitochondrial disease is of importance as it helps to identify individuals that may need changes to their care and more careful follow-up, but also because ability to identify better those with higher or lower risk of disease progression and disability may assist in directing limited healthcare resources more effectively [9, 17, 18]. In many research settings, some type of systematic and quantifiable evaluations of disability based on retrospective patient record scrutiny would also be useful, because medical notes created for clinical purposes commonly do not include such data. Rating scales specifically designed for mitochondrial disease such as the Newcastle Mitochondrial Disease Adult Scale (NMDAS) and the paediatric scale (NPMDS) are used in many dedicated tertiary care centres and for research purposes but are fairly time-consuming particularly for routine clinical care [14, 19, 20]. At present, it is not known how the more general scales for disability evaluation such as the modified Rankin scale (mRS) and the Karnofsky score scale [21–24] function in the evaluation of patients with mitochondrial disease.

We decided to investigate a single-centre cohort of patients with mitochondrial disease to evaluate whether mRS and Karnofsky scores could be determined retrospectively based on electronic patient records (EPRs), and whether these quantitative measures of functional capacity could provide insights into the risk factors related to disease progression.

## Patients and methods

In this retrospective observational study, we investigated a previously identified cohort of patients with genetically defined mitochondrial disease at Turku University Hospital (TUH, Turku, Finland). All identified patients with mitochondrial disease with available data were included. The patients were identified previously as part of a larger study on mitochondrial disease epidemiology and clinical features. The process of patient identification in this project has been recently described elsewhere [4].

Using the EPR system at TUH, we reviewed all available neurology and emergency medicine patient notes of the identified patients. Based on the EPR texts for all clinical visits, we retrospectively determined the mRS and Karnofsky scores (Fig. S1, Fig. S2) whenever the information in the texts was deemed sufficient. We also looked for the causes that lead mitochondrial disease patients to ECU visits and hospitalization. We looked in detail at whether the genetic cause of mitochondrial disease or some clinical features would be associated with the observed change in the mRS and Karnofsky scores over time.

We used the non-parametric Kruskal–Wallis test to assess differences between groups. When the overall test was significant, *post hoc* pairwise comparisons were performed using Dunn’s test with Bonferroni correction. Statistical significance was set at *p* < 0.05.

This study was approved by TUH (research permission TO4/016/16). Given its retrospective, registry-based design, individual informed consent was waived by the TUH Ethical Committee. All research was conducted in accordance with the Declaration of Helsinki.

## Results

We identified *N* = 52 patients (28 women) with mitochondrial disease whose data could be analysed. Mean age at time of data collection was 50 years (median 55 years; range 10 to 85 years) (Table 1). For one patient, no relevant data in the EPR system were found and this individual was excluded from further analysis.

**Table 1:** The patients with mitochondrial disease included in the study

Variant	m.3243 A > G	mtDNA deletion	Other mtDNA variants*	POLG variants†	Other nuclear gene variants‡	Multiple mtDNA deletions, no variant detected	All patients
Phenotype (N)	MELAS (17), MIDD (3), other (1)	PEO (4), KSS, (2), myopathy (2)	Myopathy (1), MERRF (6), PEO (1), epilepsy with SNHL (2), MELAS (2)	Ataxia, epilepsy, hepatopathy (5); PEO plus (1)	IOSCA (1), myopathy, PEO (2)	Axial myopathy (1)	
Patients, N (W/M)	21 (13/8)	8 (3/5)	12 (7/5)	6 (3/3)	3 (1/2)	1 (1/0)	51 (28/23)
Age (y) at first evaluation; mean, median (range)	45, 51 (10–72)	50, 52 (35–73)	45, 56 (17–75)	40, 38 (12–67)	42, 37 (34–56)	77	45, 51 (10–77)
Age (y) at latest evaluation; mean, median (range)	52, 56 (26–80)	59, 60 (35–77)	54, 61 (19–84)	46, 46 (20–74)	49, 43 (39–65)	85	53, 59 (19–85)

*IOSCA* infantile onset spinocerebellar ataxia, *KSS* Kearns-Sayre syndrome, *M* men, *MELAS* mitochondrial encephalomyopathy, lactic acidosis, and stroke-like episodes, *MERRF* myoclonic epilepsy with ragged-red fibres, *mtDNA* mitochondrial DNA, *PEO* progressive external ophthalmoplegia, *SNHL* sensorineural hearing loss, *W* women, *Y* years. * Including m.3260 A > G, m.3271T > C, m.4296G > A, m.7510T > C, m.8344 A > G, and m.13512G > A. † Including homozygous W748S, compound heterozygous G517V and E1143G, compound heterozygous G517V and R722H, and compound heterozygous S998L and D1184L in *POLG*. ‡ Including c.1346 C > G, p.(Thr449Arg) in *OPA1*, 39 bp duplication in *TWNK*, and homozygous c.1708 A > G (p.Tyr508Cys) in *TWNK*

The patients were divided into three groups based on the mitochondrial disease genetic background: Those with the most common mtDNA variant m.3243 A > G (*N* = 21, 13 women), all other pathogenic mtDNA variants (*N* = 22, 11 women), and those harbouring nuclear gene variants leading to mitochondrial disease (*N* = 9, 4 women) (Table 1). The average amount of data points (time points where functional status could be evaluated based on EPR notes) per patient in the EPR system was 9.3. The mean follow-up time analysed was 8.8 years (range 1 to 19 years). The group of patients with common mtDNA variant m.3243 A > G have an average of 9.2 data points, patients with all other mtDNA variants 9.9, and patients with nuclear gene variant 8.1 data points.

Few patients experienced multiple clinical events within a single age year leading to several different mRS and KPS values. The data summary includes all these multiple changes in mRS and KPS values for each age year. The mRS and KPS values determined for one data point were assumed to remain stable until different values were determined based on EPR notes.

The mean and median KPS and mRS scores and their mean change per year were calculated for the three genetic variant groups. Using the non-parametric Kruskal-Wallis test, significant differences were found between the groups (*p* < 0.01). Subsequent pairwise comparisons with Dunn’s test with Bonferroni correction revealed that the significant differences were in the first and last mRS scores, as well as the first and last KPS score, when comparing patients with mtDNA variant m.3243 A > G to the nuclear gene variants (*p* < 0.004). There was also significant difference in the first KPS score between the group including all other pathogenic mtDNA variants and the nuclear gene variants (*p* < 0.01). No other significant differences between the groups were observed (Table 2).

**Table 2:** Karnofsky performance scale and modified Rankin scale scores in patients with mitochondrial disease

Group	First KPS (%), median, mean (range 30–100)	Last KPS (%), median, mean (range 0–100)	First mRS, median, mean (range 0–5)	Last mRS, median, mean (range 1–6)	Mean change per year, KPS (%)/mRS
m.3243 A > G in mtDNA	90, 84 ^a^ (60–100)	70, 61 ^a^ (0–90)	1, 1.6 ^a^ (0–3)	3, 2.9 ^a^ (1–6)	3.6/0.3 ^a, c^
Other mtDNA variants	80, 77 ^a^ (50–100)	50, 49 ^a, b^ (0–100)	2, 2.2 ^a, b^ (0–4)	3, 3.6 ^a, b^ (1–6)	2,9/0.2 ^a, b^
Nuclear gene variants	50, 53 ^b^ (30–80)	30, 21 ^b^ (0–50)	4, 3.6 ^b^ (2–5)	6, 5.2 ^b^ (3–6)	4.9/0.4 ^b, c^
All patients	80, 76 (30–100)	50, 49 (0–100)	2, 2.2 (0–5)	3, 3.6 (1–6)	3.5/0.3

*KPS* Karnofsky performance scale, *mRS* modified Rankin scale, *mtDNA* mitochondrial DNA. KPS values (0–100%) decrease and mRS values (0–6) increase with increasing disability. For each column, groups sharing a superscript letter are not significantly different (Dunn’s post hoc test with Bonferroni correction, *p* < 0.05)

The functional capacity of the patients worsens with age in all groups. The symptom fluctuation related to clinical events such as stroke like episodes were associated with the genetic variant and phenotype. Patients with nuclear gene variants, most commonly, early-onset POLG-related disorders had fastest worsening and most severe progression per year in KPS and mRS scores and are also associated with high mortality [25]. Nuclear gene variations also presented with an early onset disease with difficult functional capacity manifestations in comparison to patients with mtDNA disease and m.3243 A > G with a phenotype of MELAS and MIDD that showed more composed decline in functional capacity throughout the follow-up period. MELAS and MIDD patients decline in functional capacity were mostly due to worsening peaks of mitochondrial disease and slowly progressive muscle weakness affecting patients’ daily motility. Patients with conditions such as chronic progressive external ophthalmoplegia (CPEO) related to mtDNA disease had a more stable disease course influenced by age of onset and the burden of mtDNA deletion [26–28]. The late-onset CPEO patients in this study align with a comparatively mild disease phenotype. Also, some of the patients with the myoclonic epilepsy with ragged red fibres (MERRF) syndrome and the Kearns-Sayre syndrome (KSS), functional capacity remained relatively stable for a long time without severe declines.

There is no clear difference between the mRS and KPS scores (Fig. S3); they tend to progress quite similarly. The scores fluctuate and the graphs draw peaks presenting worsening of mitochondrial disease often leading to ECU visits and hospitalization. Based on the EPR clinical data, the typical reasons for loss of functional capacity were stroke-like episodes, epileptic seizures, and status epilepticus most commonly associated with the m.3243 A > G in mtDNA and pathogenic variants in *POLG*. Also, fall-related events due to muscle weakness, ataxia and vertigo and infections such as aspiration pneumonia, often lead to worsening scores based on TUH EPR clinical data.

## Discussion

In this study, we retrospectively evaluated the available electronic medical records of 52 patients with mitochondrial disease to retrospectively evaluate the longitudinal progression of disability using the KPS and mRS scales. When assessing patients’ functional ability retrospectively using KPS and mRS, a decline in functional capacity over time and exacerbations of mitochondrial disease that required hospitalization, was observed across all patient groups. Our data would support the interpretation that the more exacerbations a patient experienced, the faster their functional ability declined as well as their likelihood of survival. Functional ability rarely returned to the level preceding the clinical exacerbation. The significant differences observed between the first and last mRS and KPS scores of patients with the m.3243 A > G variant and those with nuclear gene variants, and also between the first KPS of patients with other pathogenic mtDNA variants and those with the nuclear gene variants suggest that, at least in this cohort, patients with mtDNA variants in general had better functional capacity. However, our results are based on a modest cohort and therefore caution is needed to avoid overinterpretations. There is need for further studies in larger cohorts. Moreover, the use of electronic patient records to retrospectively reconstruct functional scores may introduce variability and potential bias, based on possible omissions or inaccuracies in the original notes.

Early recognition of stroke-like episodes and related seizures and aggressive initiation of treatment may help to mitigate neuronal loss and associated increase in disease burden [7, 8]. Systematic use of performance scores such as KPS and mRS may also facilitate early detection of subtle functional decline, associated with increased need of physiotherapy or walking aids, or other support of everyday living. Several features of generic functional disability scales such as the KPS and the mRS suggest that they might be useful also in the context of mitochondrial disease. One is to enable comparisons across different disorders. These scales allow standardized comparisons across diseases and populations. This is potentially useful for health economics, resource allocation, or even clinical trials where participants have diverse conditions. Multisystemic deficits have been associated with worse KPS scores in both patients with MELAS and non-MELAS m.3243 A > G patients [29]. Similarly, mRS scores increase reflecting increasing disability in adult patients with mitochondrial myopathy. This change is even more significant after stoke-like episodes [7, 28]. KPS and mRS levels have also been utilised in evaluating the functional capacity of pregnant women harbouring the m.3243 A > G variant [31]. Finally, in a study of 183 patients with mitochondrial disease, disability was assessed through in-person visits or telephone interviews using the mRS [32]. We are not aware of previous studies that have systematically presented both KPS and mRS follow-up data on a mitochondrial disease patient cohort.

Simplicity of use and accessibility are important aspects in any clinical scale. Both mRS and KPS are simple, easy to use, and require minimal training. This makes them attractive in low-resource settings or for rapid assessment during brief clinic visits. Simple scales such as the KPS and mRS can also be integrated with broader outcome frameworks. Their generic quality may also make them particularly valuable in the context of multisystem diseases like mitochondrial disorders where care by multiple speciality services is common.

Obviously, both mRS and KPS scales also have limitations. They are relatively insensitive to neuromuscular features, and neither KPS nor mRS captures symptoms that are common in mitochondrial disease such as fatigue, ptosis, muscle weakness and myopathy, and respiratory failure. These scales also lack granularity, making them somewhat insensitive especially in early stages of disease or in case of subtle symptoms. As it was originally developed for the evaluation of functional outcomes after stroke, mRS might be particularly suitable for use in the context of stroke-like episodes.

Moreover, these tools are also not ideal for tracking incremental changes, which could be of value in slowly progressive diseases. Limb muscle strength can remain quite stable, but mRS still increases, driven primarily by mortality (mRS 6) with other potential contributors of outside skeletal muscle weakness, and in other hand, patients with isolated skeletal muscle disease (CPEO) often maintain more stable mRS scores [28]. To date, there are few studies reporting the use of KPS in patients with mitochondrial disease [30].

Patients with mitochondrial disease are a highly heterogeneous group, both in terms of clinical presentation and functional capacity. The Newcastle mitochondrial disease adult scale (NMDAS) score reflects all the variations considering current function, system specific involvements, clinical assessments, and patients’ quality of life, and thus provides a broad overview of the current stage of disease [19]. For evaluating the functional capacity of children, the Newcastle paediatric mitochondrial disease scale (NPMDS) score considers also patients age, stage of development and phenotypes of early onset mitochondrial diseases [20]. KPS and mRS do not take into account the effects of younger age, severe disease forms, and developmental delay, making them potentially less applicable in the evaluation of paediatric patients. It should be stressed that neither mRS nor KPS can replace the more dedicated measures such as NMDAS in the evaluation and follow-up of patients with mitochondrial disease. However, in low-resource settings and in non-specialist care or busy clinics, they are easily applicable and give useful information on the overall function of patients. This makes these scales potentially useful complementary tools in the evaluation of the natural course and longitudinal development of mitochondrial disease.

Understanding the natural history and the risk of disability over time in various types of mitochondrial disease is of major importance for appropriate patient counselling and for the planning of clinical follow-up. Patients deemed at high risk of stroke-like episodes or refractory status epilepticus require specific attention.

This study emphasizes the importance of future investigation into preventative markers of exacerbations to improve mitochondrial disease patients’ functional capacity. Regular and systematic clinical follow-up improves our understanding of the severity and progression of mitochondrial disease. We suggest that it would be useful to incorporate the use of KPS and mRS into prospective clinical studies on mitochondrial disease, alongside dedicated scales such as NMDAS and NPMDS. In addition to the more detailed scales specifically developed for mitochondrial disease, the KPS and mRS may offer a simple and quick tool for long-term overview of the functional capacity of patients with mitochondrial disease, especially in the context of non-specialist and primary healthcare.

## Supplementary Information

Below is the link to the electronic supplementary material.

Supplementary Material 1 (PDF 389 KB)

## Data Availability

Individual patient data are not available. Aggregate original data are available from the corresponding author upon reasonable request.
