# Supplementary material for: Karnofsky performance scale and modified Rankin scale as indicators of functional capacity in patients with mitochondrial disease
Source: Neurol Sci. 2026 Jul 15;47(8):629. doi: 10.1007/s10072-026-09212-z (PMC13372874; doi:10.1007/s10072-026-09212-z)
Supplement: Supplementary file 1 — Supplementary Material 1 (PDF 389 KB) [file 10072_2026_9212_MOESM1_ESM.pdf]

**Supplementary materials**

**Fig. S1** The modified Rankin Scale (mRS).

Modified Rankin Scale (mRS)

| | |
|---|---|
| 0 | No symptoms at all |
| 1 | No significant disability despite symptoms; able to carry out all usual duties and activities |
| 2 | Slight disability; unable to carry out all previous activities, but able to look after own affairs without assistance |
| 3 | Moderate disability; requiring some help, but able to walk without assistance |
| 4 | Moderately severe disability; unable to walk without assistance and unable to attend to own bodily needs without assistance |
| 5 | Severe disability; bedridden, incontinent and requiring constant nursing care and attention |
| 6 | Dead |
| Total | 0-6 |

The modified Rankin Scale (mRS) is a commonly used clinical scale that measures the degree of disability or dependence in daily activities of people who have suffered a stroke or other causes of neurological disability. The scale range is from 0 – no symptoms at all to 6 – death.

**Fig. S2** The Karnofsky Performance Scale (KPS).

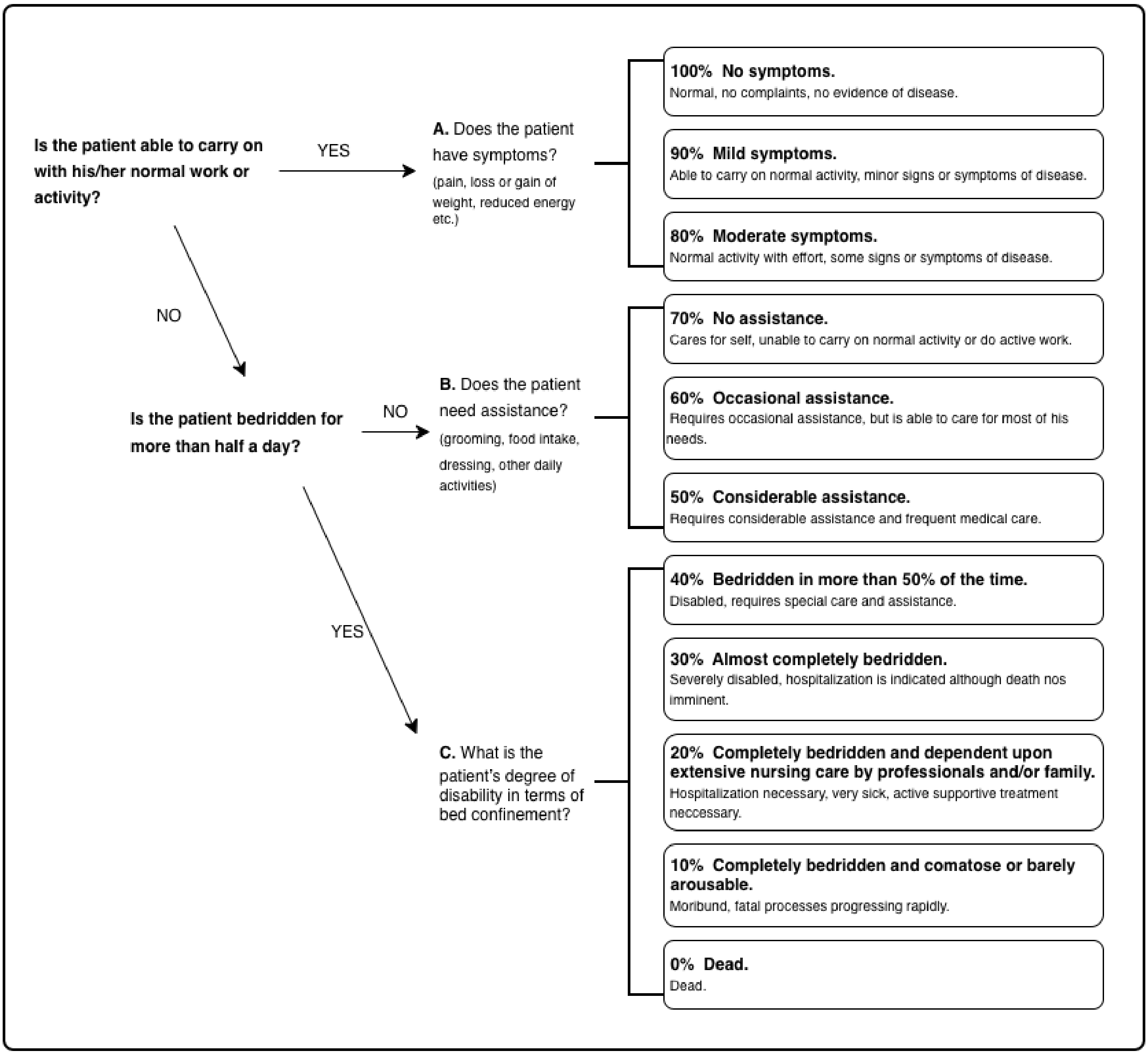


The Karnofsky Performance Scale (KPS) is a standard way of measuring a patient's functional status, especially in people with serious illnesses. It assesses how well a person can carry out ordinary tasks and helps determine prognosis, treatment options, and need for care. KPS is a numeric scale from 100% - no evidence of disease to 0% - dead in increments of 10.

**Fig. S3** Karnofsky performance scale (KPS) and modified Rankin scale (mRS) scores in patients with mitochondrial disease as a function of the time.

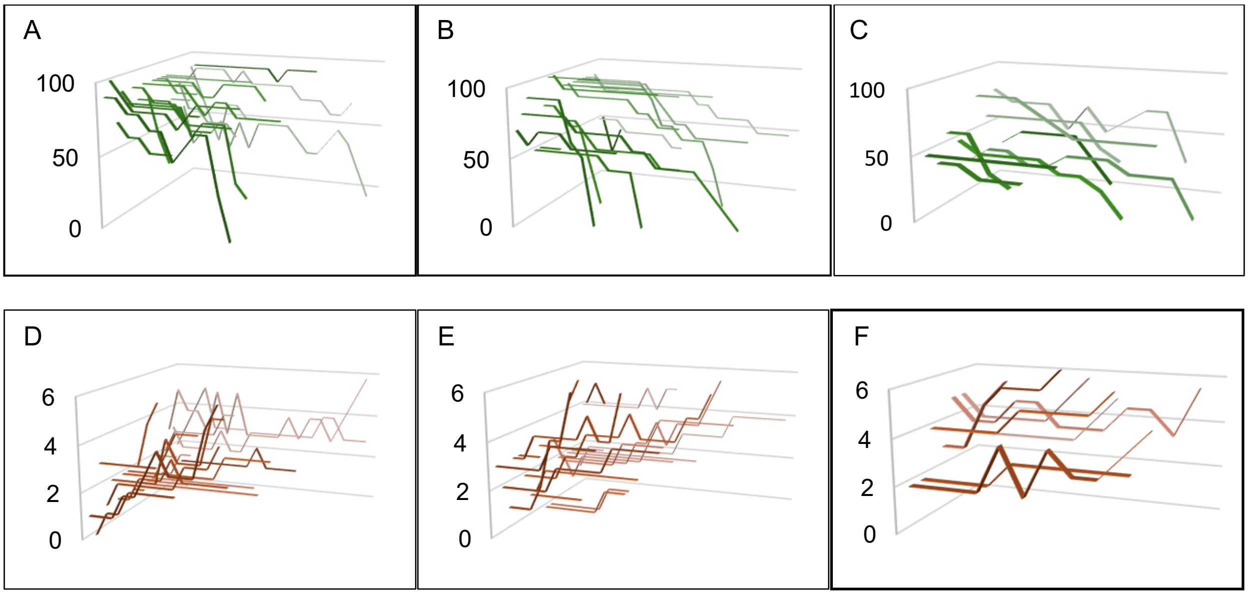


A: KPS, m.3243A>G variant in mtDNA, B: KPS, other mtDNA variants, C: KPS, nuclear gene variants, D: mRS, m.3243A>G variant in mtDNA, E: mRS, other mtDNA variants, F: mRS, nuclear gene variants
